# The collagen structure of C1q induces wound healing by engaging discoidin domain receptor 2

**DOI:** 10.1186/s10020-021-00388-y

**Published:** 2021-10-03

**Authors:** Ria Aryani Hayuningtyas, Myeonggil Han, Seoyeon Choi, Man Sup Kwak, In Ho Park, Ji-Hyun Lee, Ji Eun Choi, Dae Ki Kim, Myoungsun Son, Jeon-Soo Shin

**Affiliations:** 1 Department of Microbiology, Yonsei University College of Medicine, 50-1 Yonsei-ro Seodaemun-gu, Seoul, 03722 Republic of Korea; 2 Brain Korea 21 Project for Medical Science, Yonsei University College of Medicine, Seoul, 03722 Republic of Korea; 3 Severance Biomedical Science Institute and Institute for Immunology and Immunological Diseases, Yonsei University College of Medicine, Seoul, 03722 Republic of Korea; 4 Department of Immunology and Institute for Medical Sciences, Jeonbuk National University, Medical School, Jeonju, Jeollabuk-do 54907 Republic of Korea; 5 Department of Pediatrics, Seoul National University Boramae Hospital, Seoul National University College of Medicine, Seoul, 07061 Republic of Korea; 6 Institute of Molecular Medicine, The Feinstein Institutes for Medical Research, 350 Community Drive, Manhasset, NY 11030 USA; 7 Department of Molecular Medicine, Donald and Barbara Zucker School of Medicine at Hofstra/Northwell, Hempstead, NY 11549 USA

**Keywords:** DDR2, C1q, Collagen, Wound healing

## Abstract

**Background:**

C1q has been reported to reveal complement-independent roles in immune and non-immune cells. C1q binds to its specific receptors to regulate distinct functions that rely on the environment and cell types. Discoidin domain receptor 2 (DDR2) is activated by collagen and functions in wound healing by controlling matrix metalloproteinase (MMP) expression. Since C1q exhibits a collagen-like structure, we hypothesized that C1q might engage DDR2 to regulate wound healing and extracellular matrix (ECM) remodeling.

**Methods:**

Cell-based assay, proximity ligation assay, ELISA, and surface plasmon analysis were utilized to investigate DDR2 and C1q binding. We also investigate the C1q-mediated in vitro wound healing ability using the human fibrosarcoma cell line, HT1080.

**Results:**

C1q induced the phosphorylation of DDR2, p38 kinase, and ERK1/2. C1q and DDR2 binding improved cell migration and induced MMP2 and MMP9 expression. DDR2-specific shRNA reduced C1q-mediated cell migration for wound healing.

**Conclusions:**

C1q is a new DDR2 ligand that promotes wound healing. These findings have therapeutic implications in wound healing-related diseases.

## Background

Wound healing remains a clinical challenge, and efficient wound management is important for preventing chronic wounds. Wound healing integrates various resident and migratory cells, the extracellular matrix, growth factors, and cytokines involved in the inflammation, proliferation, and remodeling phases (Velnar et al. 2009). This complex process involving migration, proliferation, interaction, and differentiation of epidermal or dermal cells, biomolecular interactions, synthesis of matrix components occur in the injured tissue to restore wound healing (Masson-Meyers et al. 2020; Thomas 2011). Epithelial cells play a crucial role during the wound healing process by proliferating and migrating toward the injured area (Leoni et al. 2015; Landen et al. 2016). Chronic diseases such as obesity or diabetes delay wound healing, and the studies of wound assessment strategies will benefit wounded patients (Masson-Meyers et al. 2020).

C1q is the first complement component. It serves as a recognition signal that triggers the classical pathway and induces the clearance of apoptotic cells and immune complexes (Thielens et al. 2017; Trinder et al. 1996; Nayak et al. 2010). C1q consists of six trimeric globular heads (gC1q) and a collagen-like tail with Gly-Pro-Hyp collagen-like sequence repeats (cC1q) (Reid 1974). C1q in human serum binds with danger-associated molecular patterns (DAMPs) released from apoptotic and necrotic cells, including phosphatidylserine, nucleic acids, and HMGB1 (Lee et al. 2018; Son et al. 2016; Kim et al. 2018). In response to infection, C1q also binds to pathogen-associated molecular patterns (PAMPs), including lipopolysaccharides. With these features, C1q is anchored to the membrane and functions in phagocytosis, angiogenesis, apoptosis, and cytokine or chemokine induction, which are important for modulating the inflammatory response (Kishore et al. 2004; Ghebrehiwet et al. 2017). Privileged engagement of distinct C1q (gC1q vs. cC1q) regions occurs during different stages of immune cell activation and function (Thielens et al. 2017; Nayak et al. 2010). The diversity of C1q functions is related in part to its domain and depends on each receptor. There are several C1q receptors on the cell surface: (1) gC1q receptors, including the receptor for advanced glycation end products (RAGE), Siglec-3, CD91, CD33, and dendritic cell-specific intercellular adhesion molecule-3 grabbing non-integrin (DC-SIGN); and (2) cC1q receptors, including calreticulin and leukocyte-associated immunoglobulin-like receptor 1 (LAIR-1) (Thielens et al. 2017; Son et al. 2015; Son et al. 2012; Son et al. 2017). cC1q exhibits structural similarities with collagen VIII and X and the collagen receptors α2β1, LAIR-1, and DDR1 have been reported to be C1q receptors (Lee et al. 2018; Son et al. 2015).

Although several studies have elucidated the role of C1q in non-hematopoietic cells, the physiological receptor for C1q in non-hematopoietic cells and the mechanism of C1q in the wound healing process remain unclear. C1q is expressed by invading trophoblast and endothelial cells and contributes to vascular and tissue remodeling (Agostinis et al. 2016). C1q is highly expressed in the stroma and vascular endothelium in tumor microenvironments; therefore, it can serve as a cancer-promoting factor (Bulla et al. 2016). C1q also enhances endothelial cell proliferation and migration, tube formation, and angiogenesis (Ghebrehiwet and Peerschke 2004; Bossi et al. 2014; Feng et al. 2002).

Discoidin domain receptors (DDRs), including DDR1 and DDR2, are classified as receptor tyrosine kinases (RTKs) based on the presence of a catalytic kinase domain (Shrivastava et al. 1997). DDRs are activated by fibrillar and non-fibrillar collagens, which are the major components of all types of extracellular matrix (ECM), thus playing a role in the regulation of collagen–cell interactions (Marquez and Olaso 2014; Vogel et al. 1997). ECM remodeling is associated with matrix metalloproteinase (MMP) production, which is important in re-epithelialization (Marquez and Olaso 2014) as it allows for cell migration and tissue remodeling (Caley et al. 2015). Abnormalities in the healing process and dysregulation of MMPs lead to prolonged inflammation and chronic wounds. DDR2 is activated by fibrillar collagen, specifically collagen type I (Vogel et al. 1997). The catalytic tyrosine kinase domain of DDR2 undergoes phosphorylation following fibrillar collagen binding (Vogel et al. 1997). Unlike other RTKs, DDR2 phosphorylation occurs long after ligand binding and is sustained for over 24 h (Iwai et al. 2013). DDR2 is well-known for its function in wound healing. DDR2^−/−^ mice showed delayed closure of dermal wounds (Olaso et al. 2011). Furthermore, DDR2 is required for proliferative response during skin wound healing (Labrador et al. 2001).

Fibroblasts in human acute wounds express MMP2 and endothelial cells to accelerate cell migration (Giannelli et al. 1997). Likewise, MMP9 is expressed in several injured epithelia to promote wound healing (Mohan et al. 2002). Types I, II, III, IV, and V collagens activate DDR1, while types I, II, III, and X collagens stimulate DDR2 (Shrivastava et al. 1997; Leitinger 2011). Human DDR1 and DDR2 exhibit about 50% sequence homology and have a discoidin-homology domain (DD), a discoidin-like domain (DLD), an extracellular juxtamembrane domain, a transmembrane domain, a cytosolic domain, and a tyrosine kinase domain. Collagen binding to the DD induces receptor autophosphorylation (Leitinger 2003).

DDR2 signaling is required for normal bone development and wound healing, while DDR1 signaling is required for normal skeletal development, mammary gland branching morphogenesis, and blastocyst implantation (Marquez and Olaso 2014; Olaso et al. 2011; Borza and Pozzi 2014; Page-McCaw et al. 2007; Yan et al. 2014). DDR2 is also upregulated in dermal burn wounds (Feezor et al. 2004). Moreover, DDR2-deficient mice exhibit impaired dermal wound healing due to reduced fibroblast proliferation and reduced bone growth as a result of reduced chondrocyte proliferation (Olaso et al. 2011; Olaso et al. 2002).

Considering that C1q and DDRs play roles in wound healing and C1q exhibits a collagen-like structure, we hypothesized that C1q might engage with DDR2 to regulate wound healing. Here, we examined the direct binding of DDR2 and C1q and, for the first time, demonstrated the C1q-mediated DDR2 autophosphorylation and in vitro C1q-mediated wound healing ability of epithelial cells. The results would help in understanding how C1q affects the epithelial cells, and the engagement of DDR2 may contribute to the development of successful treatments to enhance wound healing.

## Methods

### Cell line and culture

The human fibrosarcoma cell line, HT1080 (ATCC CCL-121; ATCC, Manassas, VA, USA), which is epithelial-like and originated from connective tissue and preferentially expresses DDR2 mRNA and protein over DDR1 (Saby et al. 2016), was used. HT1080 cells were maintained in Minimum Essential Medium (Wellgene, Korea and Thermo Fisher Scientific, MA, USA) containing 10% fetal bovine serum and 1% penicillin–streptomycin (Wellgene and Thermo Fisher Scientific). Cell cultures were maintained at 37 °C in the humidified atmosphere of a 5% CO_2_ incubator.

### Analysis of C1q binding to DDR2 using enzyme-linked immunosorbent assay (ELISA)

Recombinant human DDR2 possessing the ectodomain of DDR2 (Gln24-Arg399) with 6 × His-tag on C-terminal (sDDR) was purchased (2538-DR; R&D Systems, Minneapolis, MN, USA). For the assay, 2 and 8 μg/mL of collagen-I (C7774; Sigma-Aldrich, Darmstadt, Germany), 2 μg/mL human C1q protein (C1740; Sigma-Aldrich), and sDDR2 were coated on ELISA plates (Corning, NY, USA) and incubated at 4 °C overnight. Bovine serum albumin (BSA; Sigma-Aldrich) was also coated as a negative control protein. The plates were washed twice with Dulbecco’s phosphate-buffered saline (DPBS; Wellgene) and blocked with 200 μL of 3% BSA at 25 °C for 1 h. Various concentrations (0.1, 0.5, 2, and 8 μg/mL) of sDDR2 or C1q were added to the wells and incubated at 37 °C for 2 h. After washing, mouse anti-DDR2 (MAB2538; R&D Systems) or rabbit anti-C1q antibody (Ab) (A0136; Dako Denmark A/S, Glostrup, Denmark) was added in 3% BSA at RT for 1 h. Horseradish peroxidase (HRP)-conjugated goat anti-mouse Ab (31430; Life Technologies, Inc., Gaithersburg, MD, USA) or goat anti-rabbit Ab (31460; Life Technologies) was used as the secondary Ab. 3,3′,5,5′-Tetramethylbenzidine (TMB) solution was added for color development, and the optical density of each well was measured at 450 nm. Non-coated blank well values were subtracted, and normalized values were subjected to statistical analysis.

### Analysis of C1qA peptide binding to DDR2 using ELISA

C1qA peptide (Biotin-GSKGEQGEPGAPGI, GenScript, Piscataway, NJ, USA), control peptide (Biotin-KAEQAEPAAPAI, GenScript), sDDR (2538-DR; R&D Systems), or BSA (Roche, Basel, Switzerland) were coated on an ELISA plate (Corning) at 8 μg/mL in PBS and incubated overnight at 4 °C. The plates were washed with 0.05% PBS with Tween-20 (PBST) twice and blocked with 1% BSA fraction V (Roche) in PBS at 25 °C for 1 h. Various concentrations of sDDR2 (0.1, 0.5, 2, or 8 μg/mL) or C1qA peptide (0.1, 0.5, 2, or 8 μg/mL) were added and incubated at RT for 2 h. After washing with 0.05% PBST, alkaline phosphatase (AP)-conjugated anti-6x-His Ab (3D5, Invitrogen, Waltham, MA, USA) for sDDR2 detection or AP-conjugated streptavidin (7105–04, Southern Biotech, Birmingham, AL, USA) for C1qA peptide or control peptide were incubated at RT for 1 h. Phosphatase substrate (S0942, Sigma-Aldrich) solution was added for color development, and the optical density of each well was measured at 405 nm.

### Surface plasmon resonance (SPR) analysis

SPR (Biacore 3000, GE Healthcare, Chicago, IL, USA) analysis was used to detect the interaction of sDDR2 with C1q proteins. With the scouted buffer conditions (10 mM sodium acetate, pH 4.0–6.0), sDDR2 protein was immobilized on the surface of the sensor chip CM5 (GE Healthcare). To derive the ligand values, a coupling process was performed based on the molecular weights of sDDR2 (MWligand) and C1q (MWanalyte). Samples were injected into running buffer consisting of 10 mM HEPES (pH 7.4) at 25 °C (10 μL/min flow rate). Sensorgrams were recorded and analyzed in real time using the Biacore 3000 control software. Six concentrations of C1q proteins (0, 2.5, 5, 10, 20, and 40 μg/mL) were used for binding to derive the K_D_ values. All exercise data were calculated using BIAevaluation Software (GE Healthcare).

### Pull-down assay

sDDR2 and C1q proteins were incubated at 37 °C for 2 h. Complete His-Tag Purification Resin (5893682001; Roche Diagnostics GmbH, Mannheim, Germany) was blocked with 3% BSA (Sigma-Aldrich) at 4 °C for 1 h. The resin was mixed with the proteins and rotated on a shaker at 4 °C overnight. After washing with 0.1% PBST, pull-down samples were subjected to sodium dodecyl sulphate–polyacrylamide gel electrophoresis (SDS-PAGE) and western blot to detect the proteins using mouse anti-DDR2 (R&D Systems) and rabbit anti-C1q Ab (Dako). For C1qA peptide and DDR2 binding assay, biotinylated-C1qA peptide (20 μg/mL) was precoated on streptavidin-conjugated microbeads (SpeedBeads™, Thermo Fisher Scientific). Beads were incubated with sDDR2 (1 or 5 μg) at 4 °C for 2 h. After extensive washing, DDR2 and C1qA peptide were visualized using western blot and dot blot with antibodies for His tag (for DDR2) or infrared 800-labeled streptavidin (for biotinylated C1qA peptide). The signals were detected by the Sapphire™ Biomolecular Imager (Azure Biosystems, Dublin, CA, USA).

### Flow cytometry

HT1080 cells were seeded in 6-well plates at a density of 5 × 10^5^ cells/mL, harvested and washed twice with DPBS, and then centrifuged at 6000 rpm for 10 min. Next, the cells were fixed with 4% paraformaldehyde at RT for 30 min and washed with DPBS twice. The fixed cells were stained with or without rabbit anti-DDR2 Ab (sc-8989; Santa Cruz Biotechnology, Dallas, TX, USA) and goat anti-rabbit Ab conjugated with Alexa Fluor 488 (A11008, Invitrogen, OR, USA) at 4 °C in the dark for 1 h. Human IgG Fc at the same concentration was used as the control. Cells were washed with DPBS and all stained cell samples were analyzed via flow cytometry using the FACSVerse system (BD Biosciences, San Jose, CA, USA).

### Confocal microscopy

HT1080 cells were seeded in 4-well chamber slides at a density of 1.5 × 10^5^ cells/well, fixed in 4% paraformaldehyde for 30 min on ice, and then washed thrice on ice with DPBS. Cells were blocked with 3% BSA for 30 min, stained with or without mouse anti-DDR2 Ab (R&D Systems) at 4 °C overnight, and then incubated with Alexa Fluor 488-conjugated goat anti-mouse Ab (A32723, Invitrogen) for 45 min in the dark. The nuclei were stained with 4′,6-diamidino-2-phenylindole (DAPI) and cells were observed with a FluoView FV1000 confocal microscope (Olympus, Tokyo, Japan).

For C1q binding assay, HT1080 cells were cultured and incubated with 2 μg/mL C1q in a serum-free medium for 2 h. sDDR2 (2 μg/mL) was also co-incubated to observe the inhibition of C1q and cell surface DDR2 binding. After treatment, the cells were washed, fixed, and blocked as described above. The cells were then stained with or without mouse anti-DDR2 Ab (R&D Systems) and with or without rabbit anti-C1q Ab (Dako) at 4 °C overnight. After they were washed thrice, the cells were incubated with Alexa Fluor 488-conjugated goat anti-rabbit Ab (A11008, Invitrogen) for C1q or Alexa Fluor 594-conjugated goat anti-mouse Ab for DDR2 (A32742, Invitrogen) for 45 min in the dark, and then mounted and examined using confocal microscopy. To determine the percentage colocalization of both proteins, images were loaded onto Image J software (NIH, Rockville, MD, USA) and the ratios of green or red to merged cells were measured using the colocalization plug-in.

### Proximity ligation assay

Proximity ligation assay (PLA) was performed according to the manufacturer’s protocol (Duolink®, Sigma-Aldrich). Cells were washed with cold DPBS, fixed with 4% paraformaldehyde for 30 min on ice, blocked with 3% BSA for 30 min, and then stained with or without mouse anti-DDR2 Ab (R&D Systems) and with or without rabbit anti-C1q Ab (Dako) at 4 °C overnight. The cells were incubated with the corresponding PLA probes conjugated to oligonucleotides (mouse MINUS and rabbit PLUS), followed by ligation and rolling circle amplification in proximity. The nuclei were stained with DAPI, and cells were observed using FluoView FV1000 confocal microscope. Interactions were quantified by counting the number of dots per cell using Image J software.

### Western blotting and immunoprecipitation

HT1080 cells were stimulated with 20 μg/mL of collagen-I, C1q (A099, Complement Technologies, Tyler, TX, USA) or C1qA peptide (Biotin-GSKGEQGEPGAPGI, GenScript) for the indicated time points. Cells were lysed in RIPA cell lysis buffer containing protease and phosphatase inhibitors (GenDEPOT Inc., Katy, TX, USA) to obtain protein extracts. Protein concentration was determined using the BCA protein assay kit (Pierce Biotech, Rockford, IL, USA). The following primary antibodies were used: mouse anti-DDR2 (MAB2538; R&D Systems), rabbit anti-DDR2 (12133; Cell Signaling Technology, Danvers, MA, USA), rabbit anti-C1q (A0136; Dako), rabbit anti-pp38 (9211S; Cell Signaling Technology), rabbit anti-p38 (9212; Cell Signaling Technology), rabbit anti-pERK1/2 (9101; Cell Signaling Technology), rabbit anti-ERK1/2 (9102; Cell Signaling Technology), and rabbit anti-β-actin (4967; Cell Signaling Technology) Abs. For western blotting, equal amounts of protein or immunoprecipitated proteins in protein sample buffer (100 mM Tris–HCl at pH 6.8, 2% SDS, 25% glycerol, 0.1% bromophenol blue, and 5% β-mercaptoethanol) were resolved by 8 or 12% SDS-PAGE and transferred to nitrocellulose membranes (Amersham Biosciences, Buckinghamshire, UK). After blocking in tris-buffered saline containing 5% skim milk and 0.1% Tween-20, the membranes were incubated with primary antibodies, followed by incubation with HRP-labeled secondary antibodies. West-Q Pico ECL solution (GenDEPOT Inc.) was used to develop the images.

To observe DDR2 phosphorylation, phosphotyrosine immunoblot was performed after DDR2 immunoprecipitation using rabbit anti-DDR2 Ab (12133; Cell Signaling Technology) at 4 °C overnight. Pre-equilibrated Dynabeads-conjugated protein G (10003D; Invitrogen) was incubated with the Ab at 4 °C for 1 h. Samples were subsequently immunoblotted by mouse anti-phosphotyrosine Ab (4G10; MP-05–321; Millipore, Burlington, MA, USA) after washing for western blotting.

### In vitro* cell wound healing assay*

HT1080 cells were grown to confluence in 12-well plates (Corning) and a straight scratch was made in each well using a 1 mL-pipette tip. The cells were then washed with DPBS twice and further cultured with collagen-I (20 μg/mL) or C1q (20 μg/mL) for the indicated time. The gap of the scratch was recorded using an Olympus IX73 inverted microscope (Olympus).

CytoSelectTM Wound Healing Assay Kit (HT1080 cells (Cell Biolabs, San Diego, CA, USA) was used (Fig. 5E and F). Briefly, HT1080 cells (3 × 10^5^ per well) were cultured in a 24-well plate until they form a monolayer around the insert that could generate a defined wound field or gap. Cells were treated with C1q (20 μg/mL; A099, Complement Technologies), C1qA peptide (200 nM) or sDDR2 (2 μg/mL; R&D Systems) in serum free X-VIVO media (Lonza, GA, USA). The wound field surface area was visualized via phase contrast or DAPI fluorescence labeling. Images were taken in three different areas per well using an Evos M7000 microscope (Thermo Fisher Scientific) at the indicated time points. The wound closure area was determined at each time point from the digital images using Image J and the wound healing rate was calculated as the (covered wound area/total surface area) × 100.

### Short hairpin RNA and transfection assay

DDR2 knockdown was achieved using stable short hairpin interfering RNAs (shRNAs). To target DDR2 (NM_006182 NCBI), we used #TRCN0000001418 (Yonsei Genomics Center System Biology Core, Seoul, Korea). #SHC016 was also used as scramble sequence shRNA. HT1080 cells were transfected using Fugene HD transfection reagent (Roche Diagnostics) according to the manufacturers’ instruction for 36 h. The knockdown efficiency was determined by western blotting using rabbit anti-DDR2 Ab (12133; Cell Signaling Technology). DDR2 knockdown HT1080 cells were subsequently plated on 12-well plates 48 h after transfection and incubated overnight. The wound healing assay was performed up to 72 h after transfection.

### Gelatin zymography

HT1080 cells were treated with collagen-I or C1q (20 μg/mL) in serum-free medium, then harvested, briefly centrifuged, and immediately frozen at − 20 °C to prevent auto-activation of MMPs. Aliquots of medium were later prepared in native buffer. Proteins were resolved by 8% polyacrylamide gels containing 0.1% gelatin without boiling. Gels were incubated in 1 × Zymogram Renaturation Buffer (Bio-Rad, Hercules, CA, USA) for 30 min, washed with distilled water, and incubated in 1 × Zymogram Development Buffer (Bio-Rad) for 30 min. Then, the gels were incubated in fresh 1 × Zymogram Development Buffer overnight at 37 °C and later stained with 0.5% Coomassie brilliant blue in 10% acetic acid and 50% methanol. The gels were destained with 50% methanol and 10% acetic acid and observed using a MiniBIS Pro imager (DNR Bio-Imaging Systems Ltd., Neve Yamin, Israel).

### Dot blot assay

Recombinant proteins (20 μg per spot), such as NS0-derived recombinant mouse DDR2-Fc chimera protein (7479-DR, R&D Systems), DDR1-Fc (6416-DR, R&D System) or Fc (4460-MG, R&D Systems) were blotted onto a nitrocellulose membrane. Both the DDR2 ectodomain (Gln24-Arg399) and DDR1 ectodomain (Met1-Thr414) fused with Mouse IgG_2A_ (Glu98-Lys330) at the C-terminal. The membrane was dried and blocked with 5% skim milk in PBS at RT for 1 h. Biotinylated C1qA peptide (200 nM) in 3% skim milk were incubated at RT for 2 h. The blots were washed thrice in 0.05% PBST and incubated with InfraRed 680 conjugated-streptavidin for 1 h. The signals were detected by the Sapphire™ Biomolecular Imager (Azure Biosystems, CA, USA).

### Quantitative real time-PCR

Total RNA was extracted from cells with an RNeasy kit (Qiagen), and cDNA was generated using an iScript cDNA synthesis kit (Bio-Rad). Real Time-PCR reactions were performed on a Light Cycler 480 II (Roche) using Light Cycler 480 Probes Master (Roche). Primers for MMP9 (Hs00957562) and HPRT1 (Hs02800695) were purchased from Thermo Fisher Scientific. The genes of interest were normalized to the housekeeping gene, HPRT1, and 2^−ΔCt^ was used to calculate the relative expression. Relative fold increase was analyzed using the 2^−ΔΔCt^ method compared to untreated control.

### Statistical analysis

Results were presented as the mean ± standard error of the mean (SEM) mentioned in each figure legend. Data were analyzed using IBM SPSS Statistics version 22.0 (IBM Corp., Armonk, NY, USA) and GraphPad Prism 9 (GraphPad Software, San Diego, CA, USA). Comparisons between more than three groups were analyzed via one-way ANOVA and the Student’s *t* test was used to compare the means between two groups. A *p* value < 0.05 was considered statistically significant.

## Results

### Binding between C1q and DDR2

**Fig. 1:**
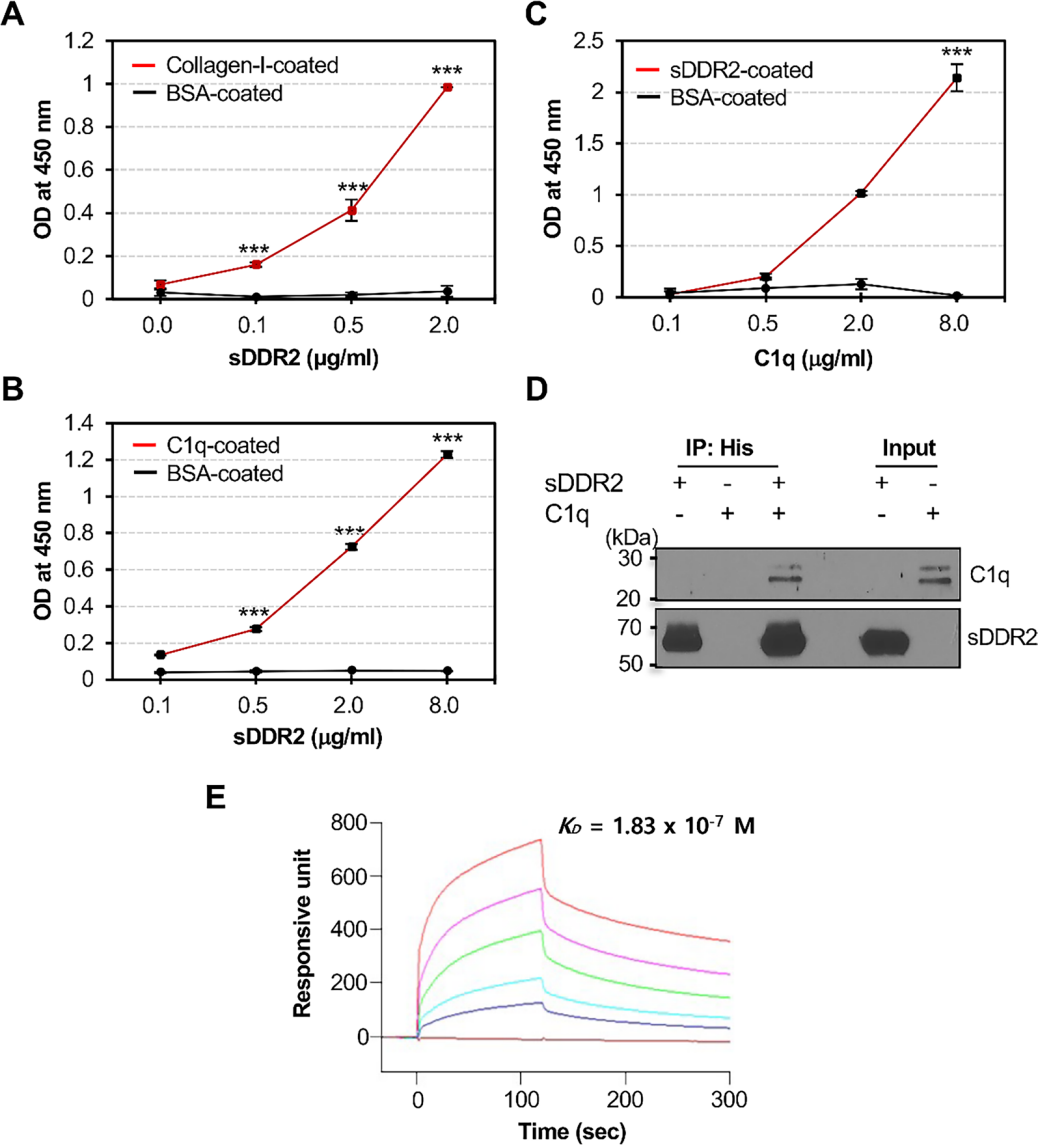
C1q binds to DDR2. **A**, **B** Microtiter plates were immobilized with 2 μg/mL collagen-I (**A**) and 8 μg/mL C1q (**B**). Respective concentrations of BSA were also coated as a negative control protein. Various concentrations of sDDR2 were added to the wells and ELISA was conducted to observe the binding. Blank well values were subtracted for analysis. **C** Microtiter plates were immobilized with 2 μg/mL each of sDDR2 and BSA, and various concentrations of C1q (0.1, 0.5, 2, and 8 μg/mL) were added to evaluate the binding of DDR2 with C1q. The graph shows the mean ± SEM of three independent experiments. *** *p* < 0.001 (one-way ANOVA). **D** Pull-down assay of 100 ng sDDR2 and 100 ng C1q were tested with Sepharose-based complete His-tag purification resin. Antibodies for western blot analyses are as indicated. **E** Surface plasmon analysis of C1q binding to DDR2. Various concentrations of C1q protein (0, 2.5, 5, 10, 20, and 40 μg/mL) were applied on an sDDR2-immobilized sensor chip CM5

DDR2 binding to C1q was examined using ELISA. As binding of collagen-I with DDR2 is well known, we tested this binding as the experiment for positive control. Microtiter wells were coated with 2 μg/mL collagen-I and BSA, and various concentrations of sDDR2 protein were added. As expected, sDDR2 bound with collagen-I in a concentration-dependent manner, while it did not bind with BSA (Fig. 1A). Next, the binding of sDDR2 with C1q was tested. sDDR2 protein was added to the microwells coated with C1q at 8 μg/mL. As shown in Fig. 1B, sDDR2 bound with C1q in a concentration-dependent manner but did not bind with BSA. In the reverse experiment, C1q was added to microtiter plates coated with 2 μg/mL sDDR2. C1q bound with sDDR2 in a concentration-dependent manner but did not bind with BSA (Fig. 1C). Next, we performed an immunoprecipitation assay to confirm the interaction between C1q and DDR2. Equal concentrations (100 ng/mL) of C1q and sDDR2 protein tagged with 6 × His were mixed and pull-down assay was conducted with His-tag-sensitive resin. C1q protein bound to sDDR2 was observed using western blotting. Two clear C1q bands (29 and 27 kDa) were observed when both proteins were present (Fig. 1D). Moreover, SPR showed the interaction of C1q with sDDR2 immobilized on the CM5 chip in a concentration-dependent manner. K_D_ value is 1.83 × 10^–7^ M, indicating that C1q has a high binding affinity for sDDR2 (Fig. 1E).

### C1q binds to DDR2 expressed by HT1080 cells

**Fig. 2:**
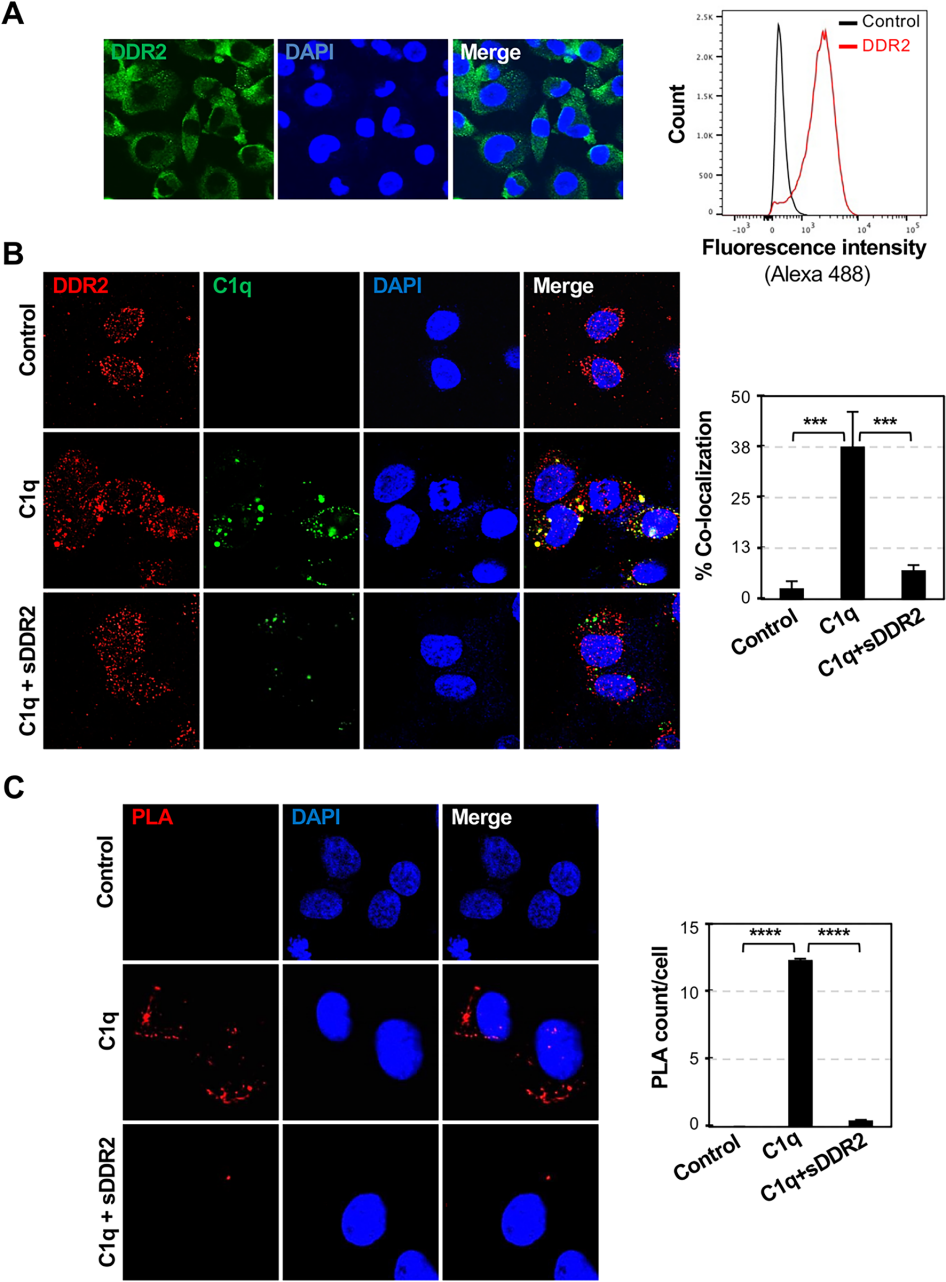
Co-localization of C1q to DDR2-expressing cells. **A** DDR2 expression in the HT1080 cell line was determined using confocal microscopic images and flow cytometric analyses. **B** Confocal microscopy analysis showing co-localization of C1q with DDR2 in HT1080 cells. The cells were treated with 2 μg/mL C1q in the absence or presence of 2 μg/mL sDDR2. Confocal images of the cells show dual staining for DDR2 (red) and C1q (green). The phenomenon was attenuated with the addition of sDDR2. **C** Proximity ligation assay (PLA) of DDR2 and C1q. The graph shows the mean ± SEM of three independent experiments. *** *p* < 0.001, **** *p* < 0.0001 (one-way ANOVA)

We observed DDR2 expression in the HT1080 epithelial cell line via confocal microscopy and flow cytometry (Fig. 2A). Next, we tested whether C1q binds to DDR2-expressing HT1080 cells by adding 2 μg/mL C1q protein to the cultures for 2 h. Then, C1q (green) and DDR2 (red) were detected using specific antibodies and the merged images were observed via confocal microscopy. C1q and DDR2 were colocalized on the HT1080 cell surface (Fig. 2B). The specificities of these interactions were further demonstrated through blocking experiments. The colocalization of C1q and DDR2 was reduced in the presence of 2 μg/mL sDDR2, confirming that C1q can bind with DDR2 (Fig. 2B). Similar results were observed in the PLA. Polymerase-amplified fluorescence showed C1q-DDR2 colocalization, and the addition of sDDR2 inhibited the C1q-DDR2 proximity (Fig. 2C).

### C1q promotes wound healing by binding with DDR2

**Fig. 3:**
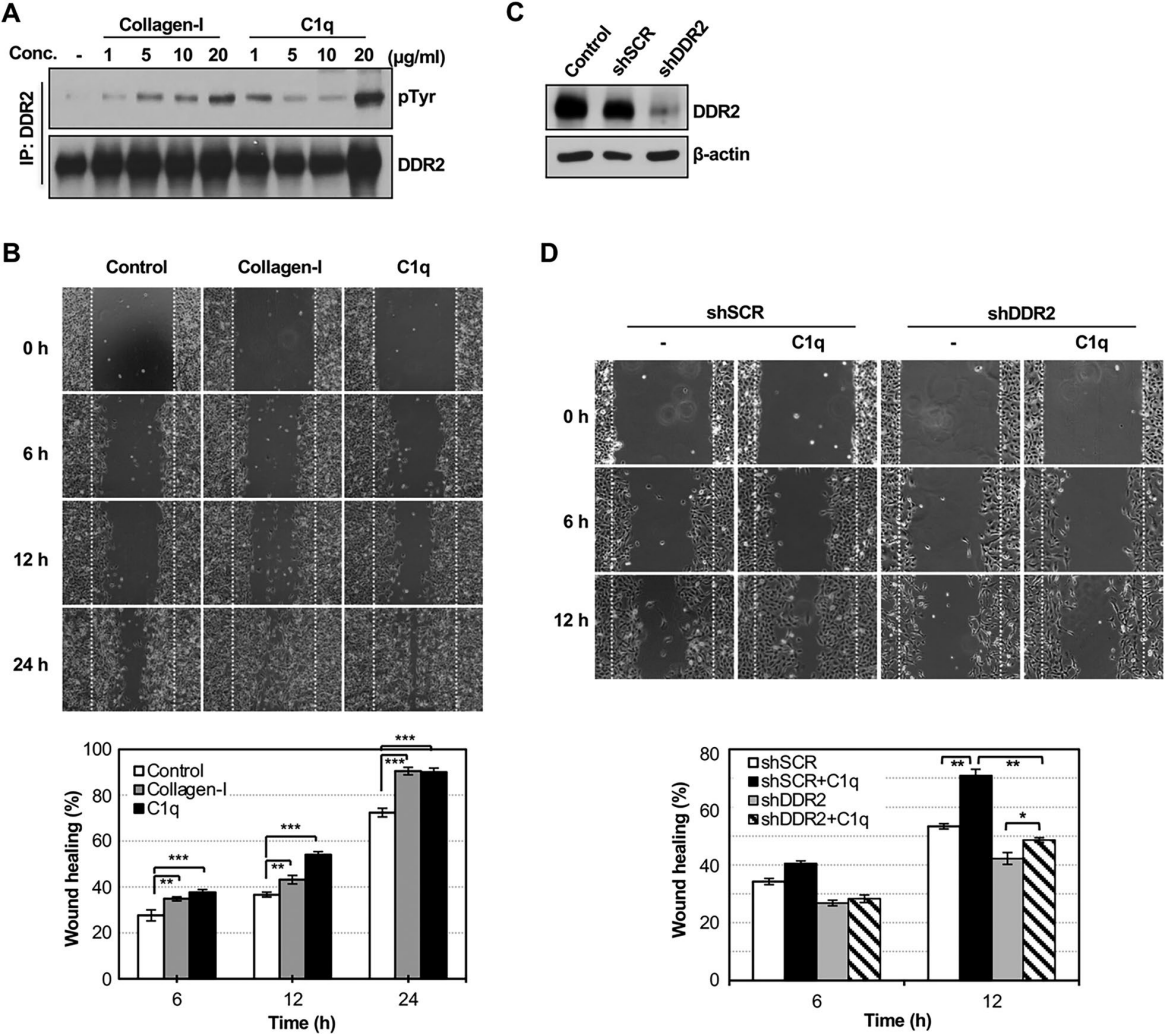
C1q-DDR2 binding promotes wound healing. **A** DDR2 phosphorylation was evaluated by immunoprecipitation with rabbit anti-DDR2 Ab and western blot with anti-phosphotyrosine Ab (4G10) or mouse anti-DDR2 Abs. HT1080 cells were incubated with the indicated concentration of collagen-I or C1q for 24 h. One representative image of three independent experiments. **B** HT1080 cells were grown to confluence in 12-well plates; a straight scratch was made and then treated with 20 μg/mL C1q for the indicated time. The wound areas were calculated and expressed as the percentage of wound closure to the initial wound areas. Values are presented as the means ± SEM of three independent experiments. **C** Western blot of DDR2 knockdown in HT1080 cells. **D** The wound healing assay was performed as described in 3B after transfection of DDR2 shRNA or scrambled control RNA (shSCR). Values are presented as the means ± SEM of three independent experiments. * *p* < 0.05, ** *p* < 0.01, *** *p* < 0.001 (one-way ANOVA)

To determine whether C1q mediates DDR2 phosphorylation, HT1080 cells were stimulated with several concentrations of C1q and collagen-I for 24 h. Then, DDR2 phosphorylation was determined via immunoprecipitation with anti-DDR2 Ab followed by immunoblot analysis for phosphotyrosine. While collagen-I phosphorylated DDR2 in a dose-dependent manner, 20 μg/mL of C1q drastically increased DDR2 phosphorylation in HT1080 cells at 24 h (Fig. 3A).

Confluent monolayers of HT1080 cells were wounded with a uniform scratch and washed to remove debris. The cells were incubated in the absence or presence of C1q for a specific duration and the wound healing rate was measured accordingly. Wound closure increased significantly with 20 μg/mL C1q treatment in a time-dependent manner (Fig. 3B).

To determine the significant role of DDR2 in C1q wound healing, DDR2 was transiently knocked down using shRNAs in the HT1080 cell line. DDR2 expression was ~ 70% downregulated by shRNA transfection at 36 h after transfection (Fig. 3C). Subsequently, DDR2 shRNA or scrambled control RNA-transfected HT1080 cells were further assessed using an in vitro scratch test for up to 72 h after transfection. C1q-induced cell migration decreased in DDR2 shRNA-transfected cells by ~ 23% compared to control cells (Fig. 3D). These data suggest that C1q-DDR2 binding induces cell migration.

### C1q induces MMP2 and MMP9 expression via p38 and ERK1/2 pathways

**Fig. 4:**
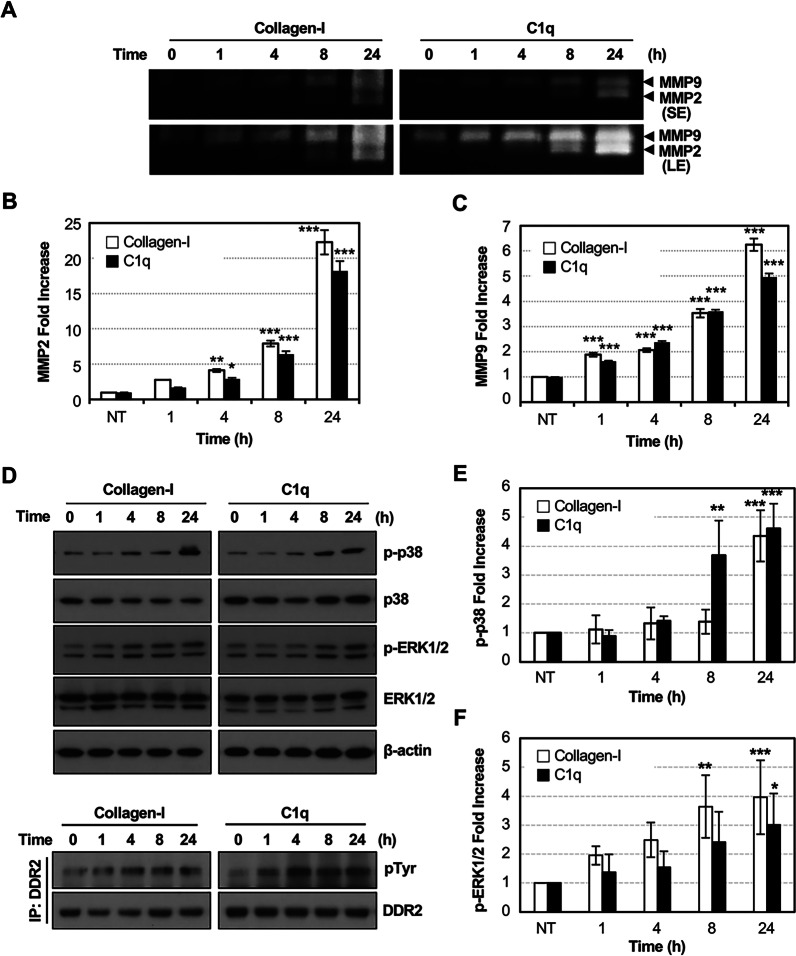
C1q induces MMP2 and MMP9 expression via p38 and ERK phosphorylation. **A**–**C** Effects of C1q on MMP2 and MMP9 production in HT1080 cells were detected via a gelatin zymography assay. The relative amounts of MMP2 (**B**) and MMP9 (**C**) were quantified using Image J. Collagen-I was used as a positive control. SE: short exposure, LE: long exposure. **D**–**F** HT1080 cells were treated with or without 20 μg/mL collagen-I and C1q for the various time points indicated. The expression of p-p38, p38, p-ERK1/2, ERK1/2, and ß-actin were detected using western blotting. DDR2 phosphorylation was tested after the immunoprecipitation of DDR2. The fold induction compared to untreated samples (NT, 0 h) was quantified using Image J. Data are presented as the means ± SEM of three independent experiments. * *p* < 0.05, ** *p* < 0.01, *** *p* < 0.001 (one-way ANOVA)

MMP2 and MMP9 productions were tested by C1q stimulation at 20 μg/mL in HT1080 cells. Gelatin zymography showed that active MMP2 and MMP9 expression were significantly induced by C1q treatment similar to collagen-I treatment in a time-dependent manner (Fig. 4A–C). Furthermore, we investigated the phosphorylation status of p38 and ERK1/2 in HT1080 cells at different time points following treatment with 20 μg/mL collagen-I and C1q to assess the related signaling pathways (Fig. 4D–F). C1q significantly increased p38 and ERK1/2 phosphorylation in a time-dependent manner for 24 h after treatment. Moreover, DDR2 phosphorylation was also continuously increased for 24 h, demonstrating that C1q is a novel functional ligand of DDR2 (Fig. 4D).

### The C1q collagen region is the DDR2 binding site

**Fig. 5:**
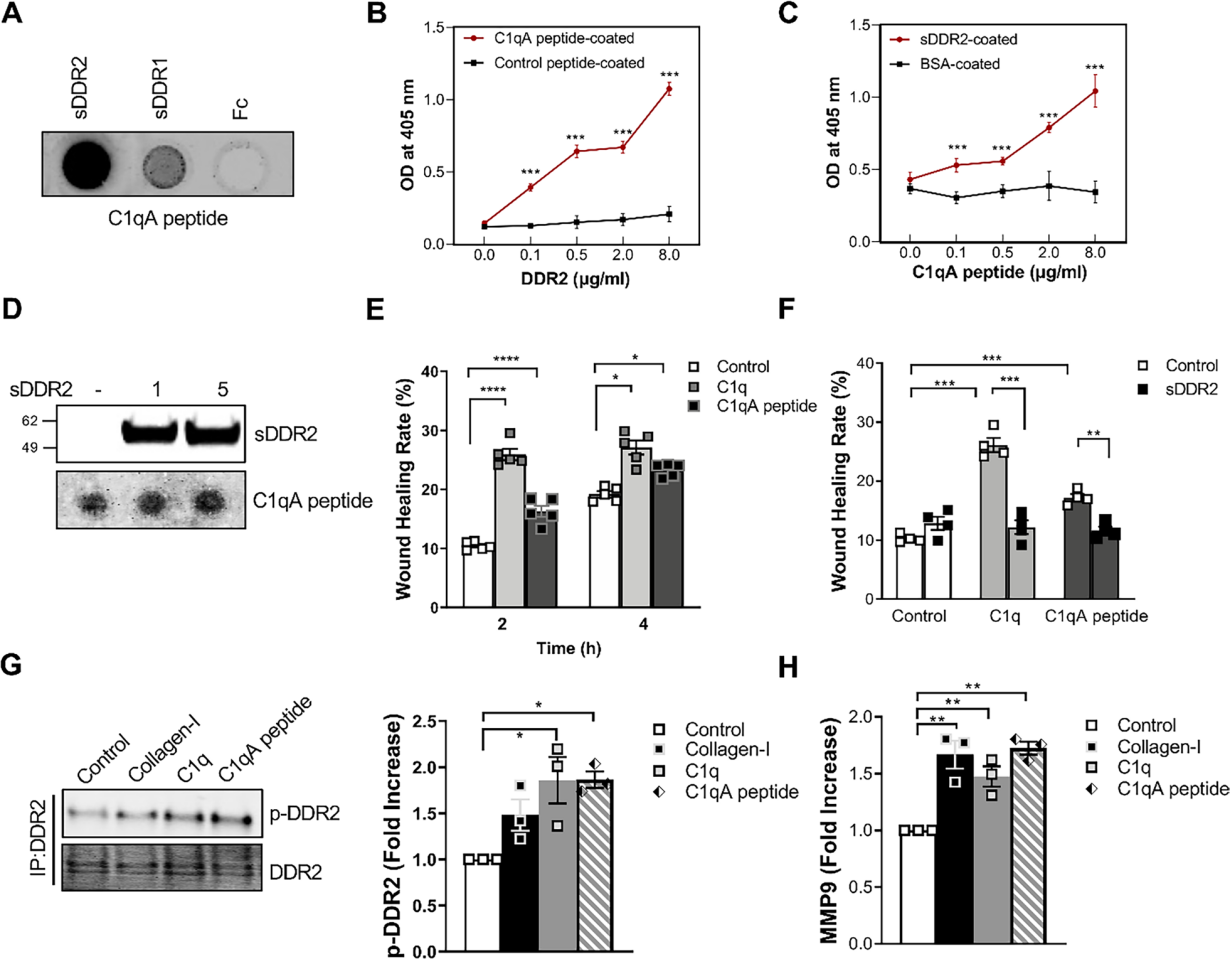
DDR2 as a receptor for cC1q in HT1080 epithelial cells. **A** C1q collagen tail peptide (C1qA peptide) binds with DDR2. Recombinant mouse DDR2-Fc chimera protein (7479-DR), DDR1-Fc (6416-DR), or control Fc (4460-MG) (20 μg per spot) were blotted onto the nitrocellulose membrane, and then detected using biotinylated C1qA peptide (200 nM). Representative image of three independent experiments. **B**, **C** Microtiter plates were immobilized with C1qA peptide or control peptide (8 μg/mL, **B**), sDDR2 (2538-DR), or BSA (8 μg/mL, **C**). sDDR2 (2538-DR; 0, 0.1, 0.5, 2.0 and 8 μg/mL, B) or C1qA peptide (0, 0.1, 0.5, 2.0 and 8 μg/mL, **C**) were added to the plates. Plate-bound peptides were detected using anti-His antibody (**B**) or AP-conjugated streptavidin (**C**). The graph shows the mean ± SE of three independent experiments. ***p < 0.001. **D** Pull-down assay of 0, 1, and 5 μg sDDR2 and 10 μg C1qA peptide-coupled streptavidin beads. C1qA peptide-bound sDDR2 was detected by anti-DDR2 Ab (MAB25381). The input of C1qA peptide was visualized by dot blot using infrared 800-labeled streptavidin. **E** Wound healing effects of C1q and C1qA peptide. HT1080 cells were treated with or without 20 μg/mL C1q or 200 nM C1qA peptide for the indicated duration. The percentage of wound closure to the initial wound areas were calculated using Image J. The graph shows the mean ± SEM of four independent experiments. **F** sDDR2 inhibits the wound healing effect of C1q and C1qA peptides. sDDR2 (2 μg/mL) was co-incubated with C1q or C1qA peptide for 2 h, and the wound healing rate was determined as described in E. Data represent the mean ± SEM of four independent experiments. * *p* < 0.05, ** *p* < 0.01, *** *p* < 0.001, ****p < 0.0001 (one-way ANOVA). **G** C1qA peptide induces DDR2-autophosphorylation. HT1080 cells were treated with 20 μg/mL of collagen-I or C1q or C1qA peptide for 24 h. DDR2 phosphorylation was determined by immunoprecipitation with anti-DDR2 Ab and western blot with anti-phosphotyrosine (4G10) Ab. The bar graph shows the mean ± SEM of three independent experiments. * *p* < 0.05 (one-way ANOVA). **H** MMP-9 mRNA expression in HT1080 cells treated as described in 5G. Data represent the mean ± SEM of three independent experiments. ** *p* < 0.01 (one-way ANOVA)

To confirm that the C1q collagen structure is a critical region for DDR2 binding, we tested whether DDR2 binds with a synthetic C1q collagen tail peptide (C1qA peptide) that is known to bind with LAIR-1 (Liu et al. 2019). We found that it binds both DDR2 and DDR1 (Fig. 5A). C1qA bound to sDDR2 in a concentration-dependent manner according to the results of ELISA (Fig. 5B and C) and pull-down assay (Fig. 5D). Furthermore, C1qA peptide promotes wound healing (Fig. 5E). The enhanced wound healing effect of C1q and C1qA peptide was diminished by sDDR2 (Fig. 5F). To observe the direct effect of C1qA peptide on DDR2 activation, we tested whether C1qA peptide can cause the autophosphorylation of DDR2. C1qA peptide induced DDR2 phosphorylation after 24 h of stimulation (Fig. 5G) and enhanced the expression of MMP-9 (Fig. 5H).

## Discussion

A complete understanding of the phases of the healing process is essential for recognizing factors that may complicate or delay wound healing (Thomas 2011). ECM remodeling is important in re-epithelialization and is associated with MMP production. Fibroblasts in acute human wounds express MMP2, and MMP9 is expressed in several injured epithelia to promote wound healing (Marquez and Olaso 2014). Studies have indicated the correlation between p38 kinase and ERK1/2 pathways with MMP2 and MMP9 activity (Yu and Kim 2016; Gweon and Kim 2013). This study shows for the first time that DDR2 is the receptor for C1q that is involved in wound healing and is present on the surface of epithelial cells. C1q and DDR2 binding improved cell migration and induced MMP2 and MMP9 expression in HT1080 epithelial cells.

Our previous data showed that C1q binds to DDR1 and promotes hepatocellular tumor progression (Lee et al. 2018). Although both DDRs are similar, they have distinct functions, expression profiles, and sequential roles in inflammation response, depending on the microenvironment and cell types (Afonso et al. 2013). DDR1 expression is increased in the glomeruli of patients with lupus nephritis (Kerroch et al. 2012). DDR2 expression increases in the cartilage of patients with osteoarthritis and correlates with the degree of cartilage damage in human knee joints (Sunk et al. 2007). Therefore, their functional differences might be related to their expression profiles and location (Abbonante et al. 2013). Moreover, other collagen-like structures containing molecules such as surfactant proteins or adiponectin may also be potential DDR ligands (Haczku 2008; Sun et al. 2016).

While C1q regulates non-hematopoietic cells such as epithelial cells, C1q also involves macrophage differentiation (Son et al. 2016; Bohlson et al. 2014). Thus, C1q may modulate infiltrated hematopoietic cells by engaging its receptors in a time-dependent manner in the wound tissue. Since C1q activates phosphoinositide 3 kinase/Akt signaling directly to DDR1 (Lee et al. 2018), PI3K/AKT might be upstream of ERK1/2 and p38. The level of C1q is increased with age and delays wound healing by modulating Wnt signaling (Naito et al. 2012). During acute wound healing, pro-inflammatory macrophages infiltrate the wound site after injury to clean the wound of microorganisms and apoptotic debris. As the tissue resolves inflammation, the overall macrophage population transitions to anti-inflammatory macrophages (Aonuma et al. 2021). Additionally, C1q could bind with the DAMP molecule HMGB1, which is secreted under proinflammatory oxidative conditions. Further study is necessary to clarify the effect of wound healing process of both molecules interacting with DDR2 (Kim et al. 2018; Kwak 2019). Thus, C1q may perform multiple functions in complicated wound regions by engaging its receptors.

Collagen peptides and LAIR-1 ligand peptides have been developed to promote cell-specific receptor functions (Liu et al. 2019; Son and Diamond 2015). According to this study, the impact of the C1qA peptide may not be limited to LAIR-1 on hematopoietic cells. DDR2 is well known to bind to the triple-helical form of collagen, and not to denatured collagen or collagen-mimetic peptides that are not triple-helical form (Carafoli et al. 2009; Konitsiotis et al. 2008). Further investigation is necessary how C1qA peptide, which does not appear to be triple-helical, bind DDR2. Our findings are potentially applicable in repurposing opportunities for treating inflammation and promoting wound healing. The present in vitro study would be helpful in preclinical models for wound healing studies (Caetano et al. 2015; Pegorin et al. 2021). Further understanding of physiological C1q binding to DDR2 is necessary for therapeutic applications in wound healing-related diseases.

## Conclusion

This work uncovers the interaction of C1q and DDR2, which promotes wound healing and possibly angiogenesis. C1q and DDR2 binding improved cell migration and induced MMP2 and MMP9 expression involved in wound healing in vitro.

## Data Availability

All data generated or analyzed during this study are included in this published article.

## Abbreviations

DDR: Discoidin domain receptor
RTK: Receptor tyrosine kinase
ECM: Extracellular matrix
MMP: Matrix metalloproteinase
gC1q: C1q globular heads
cC1q: C1q collagen tail
DD: Discoidin-homology domain
DAMP: Danger-associated molecular patterns
PAMP: Pathogen-associated molecular patterns
PRR: Pathogen recognition receptor
LAIR-1: Leukocyte-associated Ig-like receptor-1
PLA: Proximity ligation assay
RAGE: Receptor for advanced glycation end products
